# Process–Microstructure–Property Characteristics of Aluminum Walls Fabricated by Hybrid Wire Arc Additive Manufacturing with Friction Stir Processing

**DOI:** 10.3390/ma19030580

**Published:** 2026-02-02

**Authors:** Ahmed Nabil Elalem, Xin Wu

**Affiliations:** Department of Mechanical Engineering, Wayne State University, Detroit, MI 48202, USA; xinwu@gmail.com

**Keywords:** additive manufacturing, aluminum alloys, microstructure–property relationships, wire arc additive manufacturing, friction stir processing, unified additive–deformation manufacturing (UAMFSP)

## Abstract

Wire Arc Additive Manufacturing (WAAM) is a cost-effective method for fabricating large aluminum components; however, it tends to suffer from heat accumulation and coarse anisotropic microstructures, which can limit the part’s performance. In this study, a wall is fabricated using a hybrid unified additive deformation manufacturing process (UAMFSP) method, which integrates friction stir processing (FSP) into WAAM, and is compared with a Metal Inert Gas (MIG)-based WAAM wall. Infrared (IR) thermography revealed progressive heat buildup in MIG walls, with peak layer temperatures of about 870 to 1000 °C. In contrast, in the UAMFSP process, heat was redistributed through mechanical stirring, maintaining more uniform sub-solidus profiles below approximately 400 °C. Also, optical microscopy and quantitative image analysis showed that MIG walls developed coarse, dendritic grains with a mean grain area of about 314 µm^2^, whereas the UAMFSP produced refined, equiaxed grains with a mean grain area of about 10.9 µm^2^. Microhardness measurement (Vickers HV0.2, 200 gf) confirmed that the UAMFSP process can improve the hardness by 45.8% compared to the MIG process (75.8 ± 7.7 HV vs. 52.0 ± 1.3 HV; *p* = 0.0027). In summary, the outcomes of this study introduce the UAMFSP process as a method for addressing the thermal and microstructural limitations of WAAM. These findings provide a framework for further extending hybrid additive–deformation strategies to thicker builds, alternative alloys, and service-relevant mechanical evaluations.

## 1. Introduction

The Wire Arc Additive Manufacturing (WAAM) process has gained remarkable attention because of its cost-effectiveness, high deposition rate, and design flexibility. This technique is based on conventional welding processes, such as Metal Inert Gas (MIG), Tungsten Inert Gas (TIG), and Plasma Arc Welding (PAW) [1]. WAAM enables the production of large-scale aluminum (Al) based structures with minimal post-processing requirements, which makes it appealing for use in aerospace, automotive, and tooling applications [2,3]. Recently, it has been considered a sustainable alternative for subtractive fabrication methods due to its material efficiency, scalability, and potential for use in digital design processes [4,5].

Although WAAM has many advantages, it has some limitations. The parts fabricated by the WAAM process have dendritic microstructures [6,7], porosity, residual stresses, anisotropy, and coarse grains resulting from repetitive thermal cycles in its layer-by-layer deposition process [1,8]. These features compromise the mechanical performance by reducing the toughness, fatigue resistance, and isotropy of the fabricated part. Therefore, grain refinement is critical for improving the strength and durability of WAAM-fabricated parts. This could be done by reducing dislocation motion and enhancing Hall–Petch strengthening [9,10,11]. Furthermore, macrostructural distortions and residual stresses that result from excessive heat buildup during multilayer deposition can negatively affect the dimensional accuracy and fatigue performance of the components [12,13].

To address the limitations of WAAM-built parts, among the Severe Plastic Deformation (SPD) techniques, the Friction Stir Processing (FSP) is widely recognized for its ability to refine the microstructures, eliminating casting defects, and improving isotropy [11,14,15]. FSP has been shown to refine dendritic grains [6,7] into fine equiaxed structures, remarkably improving the strength and toughness of Al-based alloys [11,14,15]. The FSP process causes intense plastic deformation and dynamic recrystallization (DRX). This disrupts the columnar dendritic structures in the WAAM-fabricated parts, encourages the formation of high-angle grain boundaries, and helps mix solutes evenly. Recent studies have shown that using in situ interlayer FSP during WAAM can create a uniform equiaxed structure and decrease the grain size remarkably [16].

The interlayer FSP eliminates porosity and breaks eutectic phases, producing refined α-Al grains and increasing the tensile strength compared to that of MIG builds [17,18]. For instance, Wei et al. [18] demonstrated that applying an interlayer FSP to the WAAM 2219 aluminum alloy improved the yield and fatigue strength owing to the breakup and dissolution of coarse eutectic phases. Similarly, in Al–Cu–Mg systems, the interlayer FSP reduced porosity, refined grains to sub-5 µm, and produced nearly isotropic hardness and tensile responses [19].

Recently, a patented unified additive deformation manufacturing process (UAMFSP) was proposed. This approach directly integrates WAAM with FSP to introduce a single hybrid additive–deformation process [20]. In this method, first, the WAAM stage provides a rapid material deposition, and then the FSP stage acts as SPD on the deposited material [21] to dynamically recrystallize and refine the grains during or after deposition of each layer. This method has the potential for stabilizing the thermal cycles, suppressing the abnormal grain growth, improving equiaxedness of the grains, and enhancing the mechanical properties of the fabricated part compared with the MIG builds [3]. Recent research studies have supported this introduced concept. For instance, Yuan et al. [22] reported that the hybrid WAAM–interlayer FSP in Al–Cu alloys can form a periodic bimodal grain structure (BGS) that simultaneously enhances the strength and ductility of the fabricated part through combined discontinuous and continuous dynamic recrystallization (CDRX). Also, Guo et al. [23] presented that multiple stirring passes in the WAAM–FSP manufactured 2319 Al alloy part promoted secondary recrystallization and balanced strength–ductility relationships by reducing the coarsening of the remelted grain. These findings indicate that the controlled thermomechanical coupling via the iterative deposition and stirring can stabilize the heat-affected zone and also tailor the recrystallized grain size. Furthermore, in another study, Shan et al. [24] revealed that the addition of reinforcement particles during the FSP stage can result in dual strengthening and ductility in Al–Zn–Mg–Cu systems, thereby demonstrating the versatility of the hybrid fabrication approach.

Recent hybrid systems that combine the WAAM and FSP processes on a single platform have enabled improvement in controlling the heat flow and strain distribution. This integration reduces interlayer gradients of temperature and can promote a more uniform microstructure, leading to improved mechanical performance that approaches isotropy [25,26]. Studies on Al–Mg and Al–Si aluminum-based alloys have revealed the remarkable enhancements in thermal uniformity and refinement in microstructure [27,28]. The integration of the UAMFSP aligns with broader trends in hybrid manufacturing that merge additive, subtractive, and deformation processes for achieving improved structural integrity and dimensional precision [29,30,31].

This study investigates the process–microstructure–property relationships of aluminum 4043 walls fabricated using MIG and the patented UAMFSP processes. The analysis focuses on (i) the role of thermal accumulation during the deposition of the layers, (ii) refinement of the microstructural and statistical analysis of the grain morphology, and (iii) validation of property improvements through hardness testing. The outcomes of this research are expected to contribute to a broader understanding of hybrid process coupling and the evolution of the next-generation manufacturing technologies that enable the production of high-strength, minimal-defect, and dimensionally precise Al-based components [4,19,27,32].

Compared with previous WAAM–interlayer FSP studies that focused on microstructural refinement and bulk property changes, this work emphasizes CNC-based system integration and couples IR thermography with statistically transparent grain-morphology distributions to link thermal regime transitions to microstructural control. While interlayer FSP has been explored for grain refinement in WAAM, this study distinguishes itself by presenting the UAMFSP framework, which integrates these processes into a single, automated CNC platform. The novelty resides in the system-level coupling of real-time IR thermographic monitoring with a rigorous quantitative statistical analysis of grain morphology (area, perimeter, and roundness distributions). This approach provides a comprehensive understanding of how the transition from a melting-dominated thermal regime to a deformation-driven solid-state regime (<400 °C) enables precise microstructural control and a 45.8% increase in microhardness.

The objectives of this work are to: (i) compare the thermal behavior of MIG-based WAAM and UAMFSP using IR thermography, (ii) quantify microstructural refinement using statistical grain morphology analysis, and (iii) provide preliminary mechanical validation through microhardness measurements. The scope is limited to these measurements; tensile/fatigue testing and electron backscatter diffraction (EBSD)-based texture analysis are beyond the present study and will be addressed in future work.

## 2. Materials and Methods

### 2.1. Materials

The substrate material used in this study was an as-received commercial AA6061 aluminum alloy (purchased from Alro Metals, headquartered in Jackson, MI, USA) machined to dimensions of 152 (L) × 102 (W) × 12.7 (H) mm. The filler material used was ER4043 (an Al-Si aluminum alloy) wire (Blue Demon, Sedalia, MO, USA, 0.9 mm diameter). The nominal chemical compositions of the substrate and wire used in this study are listed in Table 1. For the deformation stage, the FSP tool was fabricated from H13 tool steel. The tool had a shoulder diameter of 18 mm and the pin diameter of 5.4 mm, with a shoulder penetration depth of 0.2 mm into the weld bead surface.

### 2.2. Fabrication Process

**Figure 1:**
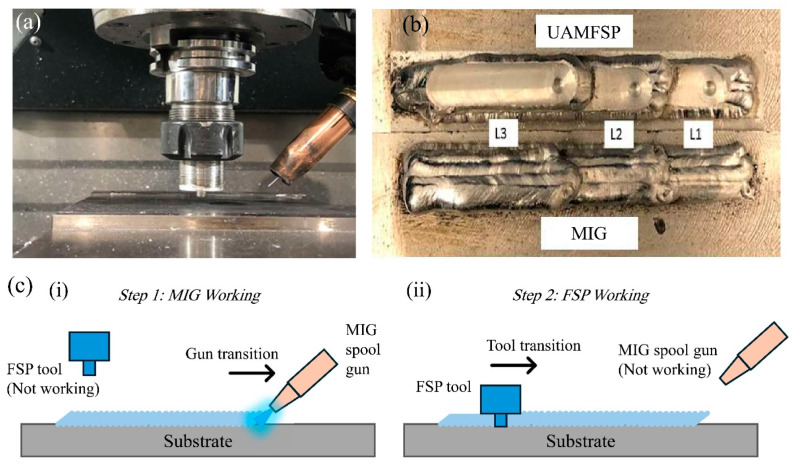
(**a**) General experimental setup showing the HAAS VF3 CNC machining center retrofitted with a spool gun for MIG deposition and equipped with an FSP tool for hybrid UAMFSP operation; (**b**) fabricated MIG and UAMFSP walls; (**c**) fabrication sequence of the walls using the UAMFSP process: (**i**) deposition sequence of MIG layers and (**ii**) execution sequence illustrating the integration of FSP.

The fabrication process of this study was performed by using a HAAS VF3 CNC machining center (Haas Automation, Inc., Oxnard, CA, USA) that was retrofitted with a spool gun connected to a power source of MIG-welding. This modification enabled an automated deposition of the bead with precise control of the welding voltage, current, wire-feed rate, and travel speed. In the hybrid process, referred to as the UAMFSP, the FSP stage was applied between layers with controlled plunge depth, tool rotational speed, and traverse speed to induce the SPD [21,33]. The experimental setup used in this study is shown in Figure 1a.

In this study, for performing comparative experiments, two types of thick walls were fabricated: a MIG-fabricated wall and a hybrid-fabricated UAMFSP wall. Each wall consisted of three layers with four overlapping beads per layer and a 50% overlap between adjacent beads. Room-temperature cooling intervals were introduced between successive layers until the interlayer temperature decreased to 38–40 °C, thereby minimizing the excessive heat accumulation [34,35]. Figure 1b shows the two types of walls, and the fabrication sequence of the walls using the UAMFSP process is illustrated in Figure 1c. For clarity, the first type of wall was produced using only the initial stage of UAMFSP, namely MIG deposition, whereas the second type of wall was fabricated using the complete two-step UAMFSP route, comprising MIG deposition followed by FSP.

The wire tip–substrate gap was fixed at 6 mm to ensure the stability of the arc. The wire feeding rate was controlled with the welding current, and pure (99%) argon was used as the shielding gas. The MIG welding process parameters are summarized in Table 2, and the FSP parameters are presented in Table 3.

Specimens were extracted from both the MIG and the UAMFSP walls for a comparative analysis. Synchronizing MIG deposition with the FSP step was challenging because of the difference in processing speeds; therefore, the second stage of the process (FSP) was initiated only after the temperature of the deposited layer decreased to 38–40 °C.

The H13 hot-work tool steel was selected for interlayer FSP because it is widely used for FSP of aluminum alloys due to its high-temperature strength, wear resistance, and chemical stability against aluminum adhesion and wear. This selection is consistent with prior interlayer FSP studies on WAAM-fabricated aluminum alloys, which commonly employ steel-based tools under comparable thermomechanical conditions [36,37]. The selected processing window (tool rotational speed 600–1200 rpm and traverse speed 50 mm/min) was chosen within literature-reported ranges for interlayer FSP of WAAM aluminum, where moderate rotation combined with low traverse speed provides sufficient severe plastic deformation and dynamic recrystallization while maintaining sub-solidus conditions. Similar settings have been used in prior studies [24,37]. The rotational speed was increased for the upper layers to compensate for the larger processed volume and increased heat dissipation with build height, thereby maintaining a comparable level of deformation and stirring intensity across layers. Because the present study used three-layer walls to enable a controlled process–thermal–microstructure comparison, direct extrapolation to taller/thicker geometries is beyond the current scope and will be addressed in future work.

The plunge depth (0.2 mm) was selected to ensure consistent shoulder engagement with the deposited bead surface while avoiding excessive thinning of the layer. The traverse speed was held constant (50 mm/min) to maintain a consistent stirring exposure per unit length, while rotational speed was adjusted (600 to 1200 rpm) across build height to maintain comparable stirring intensity under changing heat dissipation conditions.

### 2.3. Microstructural Analysis

**Figure 2:**
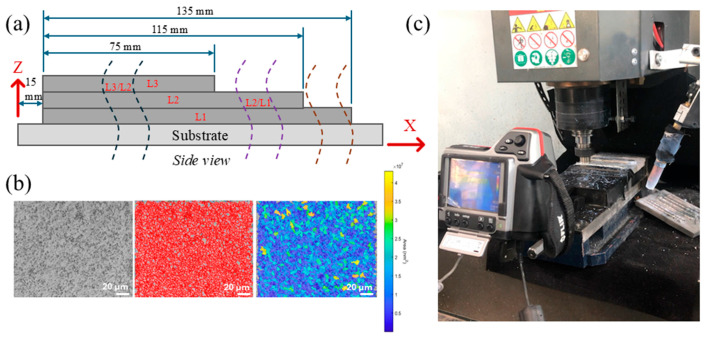
(**a**) The locations on the specimen section in the walls, (**b**) a representative MIPAR-based microstructural analysis, and (**c**) IR thermal imaging camera location in the experimental setup.

For microstructural analysis, the specimens were prepared using standard metallographic procedures, including hot mounting, sequential polishing with progressively finer abrasive papers, ultrasonic cleaning, cloth polishing with aluminum oxide powder, and final polishing with a colloidal silica suspension. The polished surfaces were etched with Keller’s reagent to reveal grain boundaries. The specimens were extracted perpendicular to the *x*-axis from each layer to obtain representative samples across the build height. The sectioning locations and orientations of the specimens are shown in Figure 2a.

The prepared specimens were first examined with a 3D laser scanning microscope (VK-9700, KEYENCE Corporation of America, Itasca, IL, USA) for evaluating the quality of their surface, followed by detailed microstructural imaging. The acquired image datasets were processed employing the MIPAR Image Analysis software module v2.x (MIPAR Image Analysis, Columbus, OH, USA) for isolating the grain boundaries and quantifying the grain metrics, like the grain area and perimeter. The outputs were then further analyzed utilizing a custom MATLAB (R2016a) script for evaluating the grain size distributions, roundness, and power-law fitting. Figure 2b depicts a representative image showing MIPAR-based microstructural analysis. Grain statistics were obtained from 27 optical micrographs per condition (9 per layer across three layers) using selected regions of interest (ROIs) that excluded free surfaces, fusion boundaries, and artifact-affected areas, yielding N = 9744 grains (MIG) and N = 10,346 grains (UAMFSP).

It should be noted that the VK-9700 (KEYENCE) is a laser confocal scanning microscope used for high-resolution 3D surface/topographical imaging; it is not a scanning electron microscope (SEM) and cannot provide compositional analysis (e.g., via energy-dispersive X-ray spectroscopy, EDS) or crystallographic orientation mapping (e.g., via EBSD).

### 2.4. Thermal Monitoring

The thermal history during the fabrication process of the walls was captured employing an FLIR T300 infrared (IR) camera (Teledyne FLIR/FLIR Systems, Inc., Wilsonville, OR, USA) to monitor and record the real-time temperature profiles. The camera provided a resolution of 76,800 pixels (320 × 240), a standard temperature range of −20 °C to 650 °C (upgraded to 1300 °C), and an accuracy of ±2%. Before use, the system was calibrated against a MIKRON M330 high-temperature blackbody source (Advanced Energy Industries Inc., Denver, CO, USA) at distances of 500 and 1000 mm, resulting in deviations of 8 °C at 1000 mm and 19–22 °C at 500 mm from the actual temperatures. These deviations were acceptable for the expected temperature range (up to 450 °C). During the experiments, the camera was positioned 500 mm from the substrate, focusing on the deposition and FSP zones in a side-view orientation (Figure 2c) and connected to a laptop running the FLIR Tools+ (v6.4) software. The temperature fields over time under the MIG and UAMFSP conditions were recorded at a frame rate of 10 fps. The software was employed for recording radiometric videos, extracting temperature–time histories, and generating radiometric images for the correlation with the microstructural analyses.

Infrared thermography requires an emissivity assumption, and for aluminum, this value depends strongly on surface condition (oxidation/roughness). During WAAM, layer-by-layer deposition promotes surface roughening and oxide formation/accumulation [38,39], and emissivity measurements on wire-arc DED aluminum parts confirm that oxidized/roughened surfaces exhibit higher effective emissivity than polished aluminum [40]. Based on the observed rough/oxidized surface condition of the as-built walls, the emissivity was set to ε = 0.95. This choice primarily affects absolute temperatures; comparative trends between MIG-based WAAM and UAMFSP are less sensitive when a constant emissivity is used [41].

### 2.5. Mechanical Testing

For evaluating the mechanical performance, the Vickers microhardness measurements were performed on the samples, employing a Tukon 2100 Micro indentation Hardness Tester (Wilson Instruments, division of Instron Corporation, Norwood, MA, USA). For the hardness measurement, the specimens were prepared according to the ASTM E3 metallographic standards [42], including sectioning, phenolic mounting, sequential grinding (240 to 1200 grit), and polishing with the diamond suspensions (6–1 µm), followed by a final polishing with 0.05 µm colloidal silica. For ensuring the integrity, the samples were cleaned ultrasonically, gently etched with Keller’s reagent, and examined under an optical microscope. The hardness measurement experiments were performed on the polished cross-sections at the ambient temperature, while maintaining a uniform spacing within the microstructural regions to ensure representative and reproducible measurements. Vickers microhardness testing was performed with an applied load of 200 gf, and for each condition, hardness was measured at 6 points (n = 6), and the reported values represent mean ± standard deviation. Microhardness (HV0.2) was measured on the cross-section at six predefined points: two in Layer 1 (center and overlap side), two in Layer 2 (center and overlap side), and two in Layer 3 (center and overlap side) for both the MIG and MIG + FSP walls.

Additional mechanical testing such as tensile, fatigue, and compression experiments was not included because the present work is scoped to a process–thermal–microstructure study on thin, three-layer walls, where microhardness mapping provides a practical first indicator of local property changes.

## 3. Results

In this section, the outcomes of comparative evaluation of the MIG and the UAMFSP walls in terms of their microstructural specifications, thermal history during their manufacturing process, and their resulting mechanical performance are presented.

### 3.1. Microstructural Analysis Results

**Figure 3:**
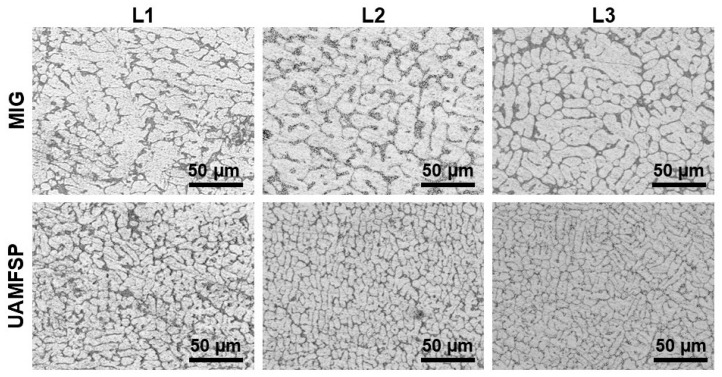
Representative optical micrographs of the MIG-deposited and the UAMFSP-processed 4043 aluminum alloy walls, illustrating the evolution of grain morphology with build height. Sequential layers (L1, L2, and L3) denote the first, second, and third layers, respectively. The images of the UAMFSP fabricated sample highlight the influence of FSP on grain refinement and uniformity of the microstructure. Scale bar = 50 µm.

As discussed earlier in the previous section, two types of thick aluminum walls were fabricated for the comparison: a MIG wall produced solely by MIG deposition and a hybrid wall produced using the UAMFSP method, which integrated MIG deposition with FSP. Quantitative analysis of the captured images was employed for evaluating the effects of the processing routes on the microstructural evolution. Representative optical micrographs of two types of walls are presented in Figure 3.

Based on the analysis, the MIG wall revealed an evolution in the grain morphology across the build height. In Layer 1 (L1) of the MIG fabricated sample, the grains were comparatively coarse, elongated, and partially dendritic, with irregular boundaries, while in Layer 2 (L2), the grains exhibited a partial refinement and spheroidization of the dendritic arms in comparison to L1, suggesting the influence of the repeated thermal cycling. In Layer 3 (L3), the progressive heat accumulation [34,35] led to a further coarsening of grains with irregular morphologies, which is consistent with the repeated reheating during the process of deposition.

In contrast, the UAMFSP wall displayed significantly more refined and homogeneous microstructures across all the layers. As can be observed in Figure 3, in layers 1 to 3, the grain morphology remained predominantly fine and equiaxed, which reflects the combined influence of plastic deformation [21,33] and localized DRX [43,44,45] induced by the FSP. The observed consistent grain size and uniform structure confirmed the strong grain-refinement capability of the UAMFSP process, which can mitigate thermal coarsening and can improve the microstructural stability.

For the quantification of the microstructural observations, the grain size and shape parameters were statistically analyzed. The analyzed outcomes are summarized in Table 4. Based on these results, the MIG wall exhibited a mean grain area of 313.6 µm^2^ with a median of 219.9 µm^2^, which confirms the dominance of large grains with a broad distribution of grain sizes. The high standard deviation of the grain area (335.2 µm^2^) indicates a significant heterogeneity in the grains of the MIG-fabricated wall. Also, the mean grain perimeter was 73.4 µm with a standard deviation of 49.6 µm, which further confirm the irregular and elongated boundary morphology of the grains. Furthermore, the mean equivalent diameter of the grains was 17.2 µm, while the mean roundness of the grains (0.71 ± 0.38) further revealed the presence of the elongated dendritic features rather than equiaxed grains.

In contrast, the UAMFSP-fabricated wall exhibited a refined and uniform microstructure. The mean grain area was 10.9 µm^2^, with a median of 6.4 µm^2^, with a much smaller standard deviation (13.1 µm^2^), which confirms a tightly clustered distribution of the grain areas. Also, the mean grain perimeter was decreased to 14 µm (σ = 9.5 µm), and the mean equivalent diameter was reduced to 3.2 µm, with a significant refinement compared with the MIG wall. Although the mean roundness (0.62 ± 0.29) was comparable to that of MIG, the reduced deviation indicated a more consistent and more homogeneous grain shape.

**Figure 4:**
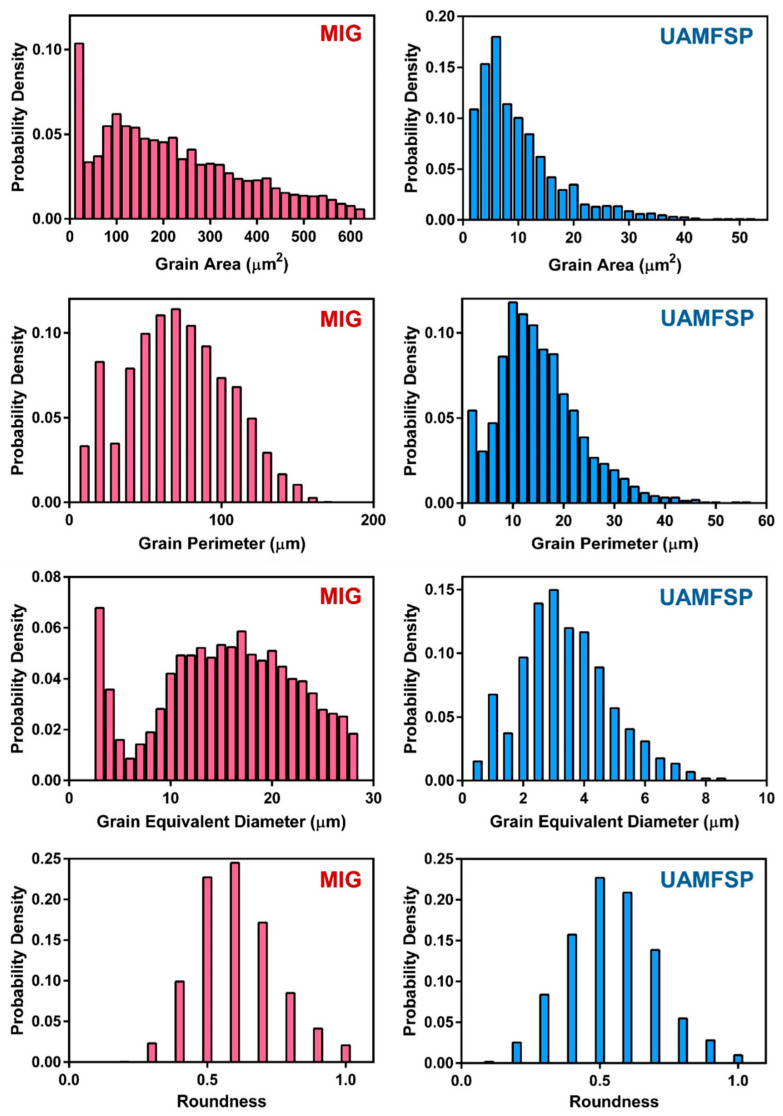
The probability density distributions of the grain area, grain perimeter, grain equivalent diameter, and grain roundness for the MIG and the UAMFSP walls. Distributions are based on N = 9744 grains (MIG) and N = 10,346 grains (UAMFSP).

**Figure 5:**
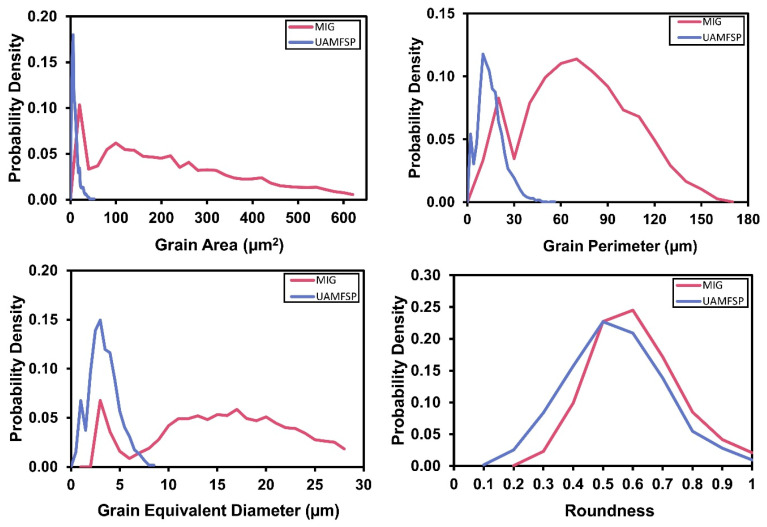
The comparative probability density curves of the grain area, grain perimeter, grain equivalent diameter, and grain roundness for the MIG and the UAMFSP walls. Distributions are based on N = 9744 grains (MIG) and N = 10,346 grains (UAMFSP).

Figure 4 shows the distributions of the probability density of the grain area, grain perimeter, grain equivalent diameter, and grain roundness for the MIG and the UAMFSP walls. Also, Figure 5 compares these distributions, which highlights the pronounced grain refinement and improved morphological uniformity that have been achieved in the UAMFSP-fabricated wall.

The presented outcomes show wide spread of elongated and irregular grains in the MIG wall. These characteristics are consistent with progressive interlayer heat accumulation [34,35], which promotes the Ostwald ripening and dendritic growth [6,7] along the thermal gradient. In contrast, for the UAMFSP wall, the narrow and uniform distributions of dense clusters of fine equiaxed grains were observed. These results, including about a 1.5-order-of-magnitude reduction in the mean grain area (from 313.6 µm^2^ in the MIG wall to 10.9 µm^2^ in the UAMFSP wall), are consistent with the role of the FSP in promoting DRX [43,44,45], fragmenting dendritic grains [6,7], redistributing boundaries, and reducing morphological anisotropy.

### 3.2. Thermal Behavior Results

**Figure 6:**
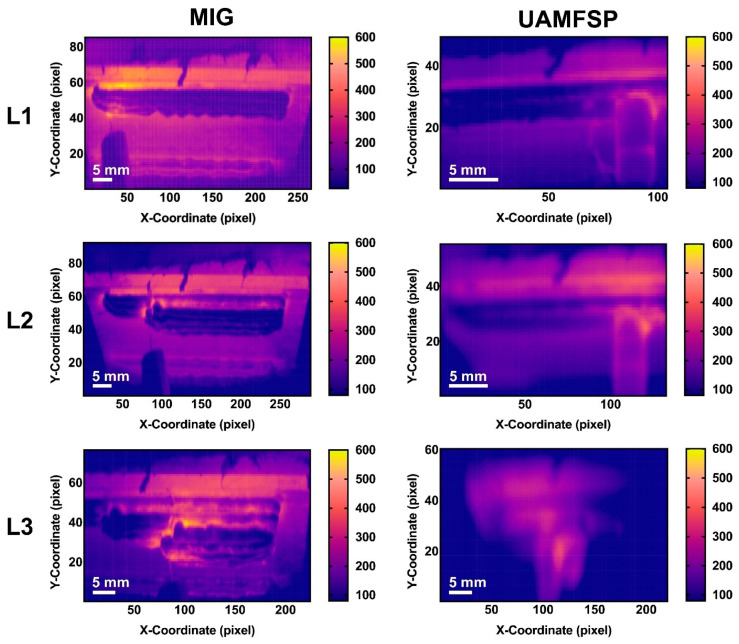
Side-view IR thermal maps; base plate at the bottom and build direction upward (+Y). The temperature contour maps (T(x,y), °C) obtained, utilizing the IR thermography, for the MIG and the UAMFSP walls across Layers 1 to 3, corresponding to L1 (60 s), L2 (50 s), and L3 (30 s). The temperature data were captured utilizing an FLIR T300 IR camera and processed in °C, with an emissivity of ε = 0.95 on the aluminum surface. The color scale represents the distribution of the spatial temperature. Scale bar = 5 mm.

**Figure 7:**
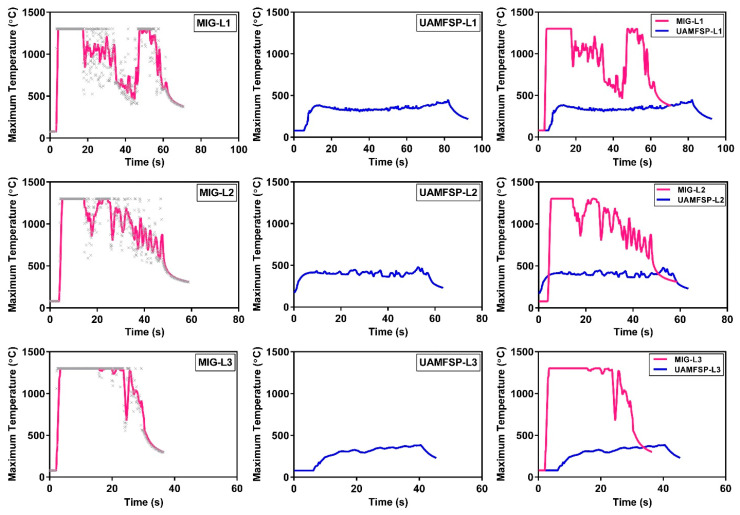
Temporal variation of the maximum temperature (°C) throughout the entire deposition and processing sequence for Layers 1–3, along with comparative results between the MIG and UAMFSP walls across the corresponding layers. Temperature data were captured using a FLIR T300 IR camera and processed in °C, with an emissivity of ε = 0.95 for the aluminum surface. Gray × markers in MIG graphs indicate the raw maximum-temperature data, and the solid pink curve shows the moving-average trend.

This section presents the thermal comparison between MIG and UAMFSP in two complementary ways: (i) spatial IR temperature fields at representative time points (Figure 6) and (ii) time-resolved evolution of the maximum temperature during each layer (Figure 7). Because IR thermography of aluminum depends on emissivity assumptions and the arc region may exhibit detector saturation, the discussion emphasizes comparative trends between the two processes and temperature evolution in the sub-saturation/interlayer regime.

The thermal evolution of the MIG and the UAMFSP walls was monitored using the infrared (IR) thermography imaging. Representative temperature contour maps obtained by IR thermography are presented in Figure 6, illustrating the spatial temperature distribution across the layers 1 to 3, corresponding to L1 at 60 s, L2 at 50 s, and L3 at 30 s after the start of each layer’s deposition or processing. As shown in the figure, the MIG wall exhibited the broad and intense high-temperature zones that expanded with the build height, that indicate progressive interlayer heat accumulation. In contrast, the UAMFSP wall maintained the uniformly distributed low-intensity contours, that reflect superior thermal management and minimal residual heating.

As can be observed, Figure 6 reveals that the layers in the MIG wall that are shown in the left column have a higher maximum temperature with a broader distributions. The peak values reach to approximately 600 °C in layers 1 to 3. This indicates substantial heat accumulation and a reduced cooling efficiency along the successive depositions in the MIG wall. On the other hand, the layers in the UAMFSP wall, that are shown in the right column, maintained lower and more uniform temperature fields, with the peak temperatures of about 300 °C across all layers, reflecting efficient thermal dissipation and localized heat generation in the interface of the tool and the sample. This comparison highlights that the UAMFSP process can significantly reduce the overall heat input and can mitigate interlayer thermal buildup, compared to the conventional MIG deposition. This reduction in the maximum and residual temperatures can improve the microstructural uniformity and prevent the defects that could be induced by overheating. These results confirm the capability of hybrid deposition–deformation strategies in redistributing the heat and mitigating the thermal gradients [21,33]. The distinct temperature regimes align with the refined grain structures observed in Section 3.1, that confirm the reduced thermal exposure in the UAMFSP process can promote stable and fine-grained microstructures.

As the temperature contour maps shown in Figure 6 represent the instantaneous thermal states at selected time points, they do not capture the complete temporal evolution of heat accumulation during the whole process. To address this limitation, the variations in the maximum temperature with time throughout the entire deposition and processing sequence for layers 1 to 3, in both the MIG and UAMFSP walls, are presented in Figure 7. As shown in this figure, the MIG condition exhibited sharp, repeated temperature spikes corresponding to arc ignition and droplet transfer events, followed by brief cooling intervals between the passes. The observed fluctuating temperature profile for the MIG wall indicates an unstable heat input and significant thermal cycling, which is consistent with the melting–solidification behavior of the MIG process during the welding. In contrast, the UAMFSP condition was demonstrating a much smoother and more stable thermal curve, with the peak temperatures being maintained at around 300 to 400 °C for all the layers. The absence of abrupt spikes confirms that the heat generation in the UAMFSP wall is governed mainly by frictional and plastic deformation mechanisms rather than the melting. This steady-state temperature evolution reflects effective heat redistribution and rapid dissipation during the hybrid processing, which minimizes interlayer thermal gradients and prevents excessive local heating.

As shown in Figure 7, for the MIG wall, the thermal profiles, which were recorded by the IR camera, occasionally displayed clipped peaks near the maximum measurable temperature of 1300 °C. Detector saturation can occur when the scene temperature exceeds the camera measurement range, causing the hottest pixels near the arc/melt-pool region to clip and reducing confidence in absolute peak temperatures. Accordingly, the reported peak values in saturated frames should be interpreted as lower bounds, and our discussion emphasizes (i) relative comparisons between WAAM and UAMFSP under identical settings and (ii) temperature evolution in the sub-saturation regime (e.g., interlayer/solid-state regions used to interpret thermal accumulation and process transitions). High-dynamic-range approaches are one possible route to capture wider temperature spans in future work [41]. This saturation suggests that the localized regions (particularly within an arc core and molten droplets) temporarily reached temperatures higher than the calibrated range. This transient overheating is characteristic of the aluminum 4043 MIG welding, where the arc plasma and the molten pool can reach into highly elevated temperatures [46]. Although these extreme values were not fully captured, the clipped peaks on the plots confirm their occurrence. However, this limitation does not affect the comparative analysis as the UAMFSP condition peak temperatures remain within the solid-state temperature range, that ensure a reliable differentiation between the melting-based MIG and FSP processes.

**Figure 8:**
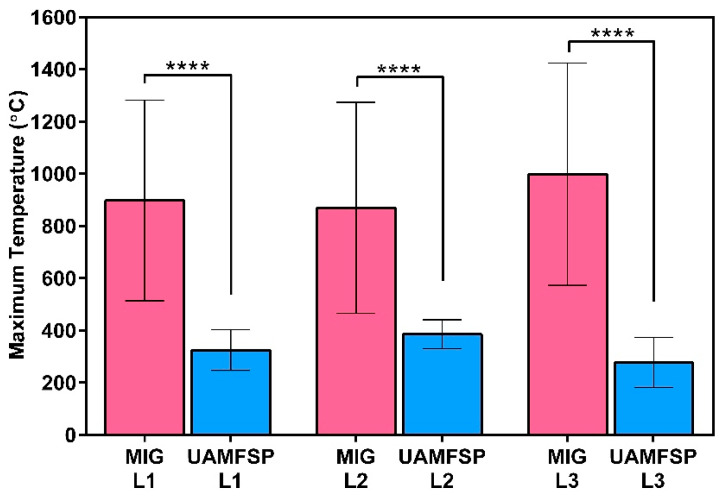
The comparison of the maximum temperatures (mean ± SD) measured at Layers 1–3 for the MIG and UAMFSP walls. The UAMFSP process exhibited significantly lower temperature peaks across all layers (**** *p* < 0.0001).

Figure 8 and Table 5 show a comparison of the maximum temperatures measured through layers 1–3 for the MIG and UAMFSP walls. The results demonstrated that the MIG process generated significantly higher peak temperatures at all layers than the UAMFSP process. Specifically, the mean maximum temperatures for the MIG ranged from 869.4 °C to 998.2 °C, whereas the UAMFSP showed much lower temperatures ranging from 277.2 °C and 386.2 °C. Based on the *t*-test analysis, this difference was statistically significant for each layer (**** *p* < 0.0001)). The standard deviations were also remarkably higher for the MIG wall (384–426 °C) than for the UAMFSP wall (55–96 °C), indicating higher thermal fluctuations and non-uniform heating during conventional MIG deposition. In contrast, the temperature in the fabrication of the UAMFSP wall had more stable and controlled conditions, which can be helpful for decreasing residual stresses, reducing microstructural heterogeneity, and decreasing potential metallurgical defects in the fabricated wall. The results revealed that the integration of the FSP stage in the UAMFSP process effectively suppressed excessive heat accumulation and promoted a more uniform thermal profile, contributing to improved process stability and microstructural refinement compared with conventional MIG deposition.

### 3.3. Mechanical Properties Results

**Figure 9:**
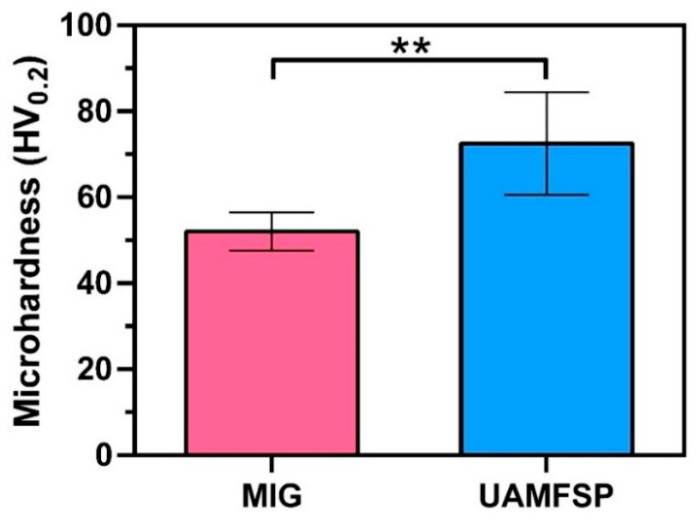
The Vickers hardness (HV0.2) measurement results of the MIG and the UAMFSP fabricated samples. Error bars represent one standard deviation. The double asterisk (**) indicates a statistically significant difference (*p* = 0.0027).

The mechanical responses of the two walls were evaluated through the Vickers microhardness testing (HV0.2), following the procedure described earlier. Figure 9 presents graphically the measured mean hardness values and their corresponding standard deviations for the MIG and the UAMFSP conditions. As can be seen in this figure, the MIG wall exhibited an average hardness of 52.0 ± 1.3 HV, indicating a relatively low strength, consistent with the aluminum structures fabricated by the conventional wire-arc deposition. In comparison, the UAMFSP wall achieved a hardness of 75.8 ± 7.7 HV, corresponding to an increase of 45.8% compared to the MIG wall. Statistical analysis (*t*-test, *p* = 0.0027) confirmed that this improvement in the UAMFSP wall was statistically significant compared to MIG wall, as indicated by the double asterisks in Figure 9.

Similar improvements in hardness through grain refinement have been reported in aluminum alloys in previous studies, where Hall–Petch strengthening dominates the structure–property relationship [9,10,47]. The enhancement in the hardness of the UAMFSP wall reflects the combined effects of the fine equiaxed grains, the reduced morphological anisotropy, and the enhanced boundary density that were produced by the DRX [22,25,48].

Although the UAMFSP wall displayed a slightly higher scatter in the hardness values, this variability remained within the typical range expected for the hybrid additively processed aluminum components. Overall, the UAMFSP condition exhibited consistently higher hardness across the measured regions compared to the MIG wall. A detailed interpretation of the strengthening mechanisms and their relationship with the refined microstructure is provided in the Section 4.

## 4. Discussions

### 4.1. Microstructural Analysis Discussions

The earlier presented microstructural analysis results emphasize the important role of the processing strategy in controlling the microstructure. The contrast in the evolution of the microstructures between the conventional MIG-deposited wall and the hybrid UAMFSP wall highlights the mechanisms governing grain coarsening, refinement, and the recrystallization in these two processes.

For the MIG wall, the progressive coarsening quantified in Table 4 and shown in Figure 3, along with the broad statistical spread reported in Table 4 and Figure 4 and Figure 5, can be attributed to the interpass heat accumulation [34,35] during the successive deposition of the layers. The repeated reheating of already solidified layers encourages Ostwald ripening and dendritic grain alignment [6,7] through the prevailing thermal gradient, resulting in anisotropic and heterogeneous microstructures. A similar behavior has been reported in WAAM-fabricated aluminum alloys, where heat accumulation causes abnormal grain growth and lower structural uniformity [34,35]. The presence of coarse, irregular grains in the MIG-deposited wall can increase the likelihood of the formation of a stress concentration site, thereby reducing fatigue resistance and declining the overall mechanical reliability [6,7]. The heterogeneous structure of the MIG wall, characterized by a mean grain area of 313.6 µm^2^ and a high standard deviation of 335.2 µm^2^, implies a strong spatial variation in the local solidification rates, in line with the reports on WAAM aluminum alloys, where the variable thermal gradients lead to uneven grain morphologies and the solidification behaviors [49]. These coarse, irregular grains are likely acting as the stress concentrators under the cyclic loading, which can reduce the fatigue strength and the reliability [50,51]. Therefore, the MIG microstructure typifies a thermally dominated WAAM system in which high peak temperatures and thermal cycling govern the grain coarsening.

In contrast, the UAMFSP wall exhibited comparatively refined, equiaxed, and more homogeneous grains across all build layers, as shown in Figure 3. The outcomes revealed that the average grain area was decreased by about 1.5 orders of magnitude (from 313.6 µm^2^ to 10.9 µm^2^) compared to that of the MIG-deposited wall. This improvement is consistent with the mechanisms of the FSP, where SPD [21,33] thermal stirring fragments dendritic grains [6,7] and drives dynamic recrystallization (DRX) [43,44,45,48,52]. The tight statistical distributions and equiaxed morphologies validate that the FSP stage in the UAMFSP process can help to mitigate anisotropy and achieve uniform grain refinement, which is consistent with previous studies on hybrid additive–deformation processes [21,33]. Additionally, the combination of moderate thermal input (300 to 400 °C, as shown in Figure 8) and rapid heat dissipation prevents the abnormal grain growth, which produces stable subgrain structures with high-angle boundaries [48,52,53]. This microstructural stability can explain the narrow statistical spread of the grain size (σ = 13.1 µm^2^) and perimeter (σ = 9.5 µm) in the UAMFSP fabricated wall. The observed more homogeneous microstructure and grain uniformity suggest that the thermomechanical environment during the FSP stage of the UAMFSP process can effectively mitigate the thermal gradients while maintaining a controlled deformation-driven recrystallization regime. These results agree with those of hybrid FSP-assisted WAAM systems reported for aluminum alloys [19,22,25,54,55,56,57,58,59], in which post-deposition or interlayer FSP reduced porosity, homogenized solute distribution, and significantly refined the structure of the grain.

**Figure 10:**
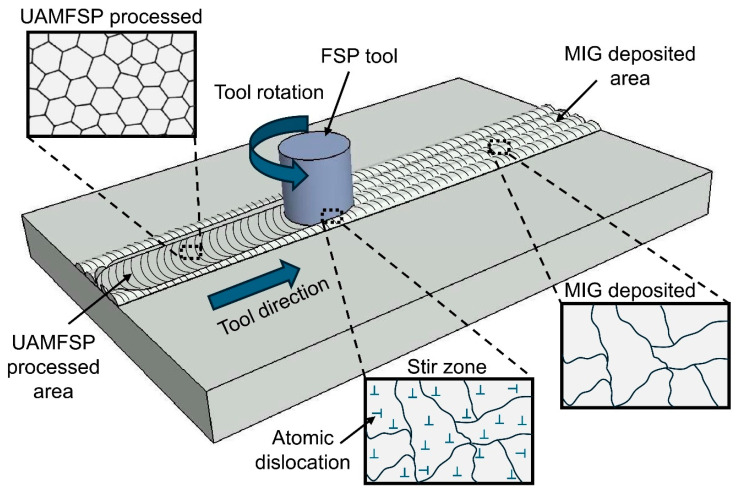
Mechanism of grain refinement in UAMFSP via CDRX. The rotating FSP tool refined the MIG-deposited layer (bottom-right inset: dendritic grains) into fine equiaxed grains (top-left inset).

The DRX during the FSP stage of the UAMFSP process can introduce the high-angle grain boundaries that act as dislocation barriers, and frictional heat exposure below the solidus temperature promotes fine sub-grain formation without coarsening. These mechanisms collectively stabilize the dislocation structures and can improve the strain accommodation. The intimate coupling of the frictional heating and plastic flow can create the favorable conditions for CDRX, leading to refined, equiaxed, and defect-free grain structures. Figure 10 schematically depicts the grain refinement mechanism in UAMFSP via CDRX. The rotating FSP tool refined the MIG-deposited layer into fine, equiaxed grains.

The achieved grain refinement ratio (~29× decrease in the mean grain area) is consistent with the reported DRX refinement factors for FSP-processed aluminum alloys [54]. Furthermore, the observed homogeneity across layers 1–3 indicates that the UAMFSP overcomes the common limitation of WAAM, namely, the microstructural gradient along the build direction [22]. Similar hybrid approaches, such as interlayer hot rolling [60] and FSP [56], applied to WAAM deposits, have also demonstrated strength improvements and microstructural/mechanical homogenization by controlling the thermomechanics of their relevant process. Therefore, the present study confirms that integrating a solid-state deformation step directly after deposition is an effective strategy for stabilizing the microstructure and enhancing the structural integrity of aluminum alloy WAAM components.

Because EBSD/texture analysis was not performed in this study, the presented results support morphological/behavioral uniformity, including grain-shape statistics and hardness values rather than definitive crystallographic isotropy. Prior WAAM + FSP studies that include EBSD show that stirring with severe shear deformation and DRX can alter grain orientation and texture strength; however, the resulting preferred orientation is process/sequence-dependent and should therefore be verified by EBSD in future work [53,58].

### 4.2. Thermal Behavior Discussions

The thermal profiles, that were presented in Section 3.2, highlighted the divergent heat management between the MIG and the UAMFSP walls. In the MIG condition, progressive heat accumulation [34,35] was observed in each layer. Such accumulation is characteristic of WAAM processes without interpass cooling, where a high heat input promotes reduced cooling rates and localized reheating [34,35]. The IR thermography results showed broad, intense high-temperature zones extending through the wall height, with mean peak temperatures between 869 and 998 °C and standard deviations of approximately 384–426 °C, as shown in Table 5.

The microstructural transformations discussed earlier correlate with the distinct thermal history of the two processing routes. The MIG wall experienced extreme and fluctuating maximum temperatures between about 900 to 1000 °C, occasionally exceeding the calibrated limit of the IR camera, that was about 1300 °C, whereas the UAMFSP wall maintained stable peak temperatures below 400 °C (Figure 7 and Figure 8). This reduction in the temperature amplitude minimizes solute diffusion and grain coarsening kinetics [61]. Figure 8 confirms that the peak temperature is lower than 400 °C, which is fairly below the Al-Si eutectic (~577 °C) [62], which can prevent remelting and preserve the solid-state nature of the FSP. The lower and more uniform heat input during the FSP stage of the UAMFSP process enhanced heat dissipation through the substrate and tool shoulder, reducing the residual heat and suppressing the epitaxial grain growth.

Occasional clipped peaks near the IR camera limit of about 1300 °C indicate transient local overheating during arc ignition and droplet transfer, which is a known feature of aluminum MIG deposition [46]. Such thermal nonuniformity produces steep gradients along the build direction, which provides a driving force for anisotropic grain growth via dendritic alignment [6,7]. This mechanism explains the anisotropic coarsened morphologies observed in Figure 3 and is consistent with prior studies on the WAAM process that link the excessive interpass temperatures to the abnormal grain growth, residual stresses, and microstructural heterogeneity [58].

The UAMFSP wall exhibited peak temperatures below 400 °C with the uniform contours and moderate cooling rates throughout all layers. Mechanical stirring during the FSP stage of the UAMFSP process redistributes the heat, limits the extreme gradients, and prevents the localized overheating. The friction-stir-caused convection can enhance the heat dissipation through the shoulder and substrate, which maintains the material well below the melting range, and prevents the interlayer thermal buildup. The lower and more uniform temperatures (mean approximately 277–386 °C with standard deviations 55–96 °C) confirm the stabilized thermal regime achieved through the FSP stage in the UAMFSP process. This steady-state heating mode helps minimize the residual stresses, mitigating the thermal gradients, and providing favorable conditions for CDRX, accounting for the refined equiaxed grains observed in the Figure 3. The stark contrast between these two regimes of the temperature underscores the fundamental differences in the mechanisms of heat generation. Similar redistribution mechanisms have been reported in the hybrid additive–deformation processes [48,52,63], where SPD [21,33] reduces thermal accumulation and stabilizes microstructural development.

MIG deposition is dominated by the arc-based melting and solidification, which can produce sharp thermal spikes and steep gradients, whereas the FSP stage in the UAMFSP process is based on distributed frictional and plastic work heating, which generates a lower-magnitude, spatially uniform temperature field. Consequently, the UAMFSP process integrates both thermal and mechanical stabilization, ensuring a controlled solid-state thermal cycle that can suppress overheating and enhance the reproducibility of the process. These results are consistent with those of earlier hybrid-WAAM investigations [52], which demonstrated that the active control of the interlayer temperature effectively improves the microstructural uniformity, hardness, and the overall build integrity.

### 4.3. Mechanical Properties Discussions

The outcomes of microhardness measurements further validated the microstructural differences between the MIG and the UAMFSP walls. Grain refinement plays a crucial role in the mechanical performance of the metallic materials. According to the Hall–Petch relationship, the yield strength increases inversely with the square root of the grain size, that indicates the smaller grains can provide higher resistance to the motion of dislocation along the grain boundaries [64]. In this context, the substantial grain size reduction achieved through the UAMFSP process led to a notable improvement in the yield strength compared to that of the coarse-grained MIG-deposited wall. As the hardness value is directly correlated to the yield strength, this microstructural refinement also manifests as a measurable increase in hardness values, which confirms the strengthening effect of fine equiaxed grains produced by DRX [64].

The MIG wall, with the measured hardness of 52.0 ± 1.3 HV, displayed a comparatively low value of hardness that confirms the coarse dendritic microstructures [6,7] and residual anisotropy typical of aluminum components fabricated by the conventional WAAM method [9,10,47]. The limited grain boundary density and the presence of elongated, irregular grains (as can be observed in Table 4) reduced the effectiveness of dislocation hindrance, that lead to a lower hardness and strength. This behavior aligns with earlier reports that insufficient thermal control during arc-based deposition produces heterogeneous microstructures with localized stress concentrations, which then diminish the mechanical efficiency and fatigue reliability [59].

On the other hand, the UAMFSP sample exhibited hardness values of 75.8 ± 7.7 HV, corresponding to an increase of ~45.8% compared with the MIG condition (*p* = 0.0027; Figure 9). This improvement can be mainly attributed to Hall–Petch strengthening, whereby the finer grain sizes (from ~17.2 µm to ~3.2 µm) increase the resistance to dislocation motion [9,10,47]. Although the UAMFSP wall displayed slightly higher scatter in the hardness values, this variation remained within the typical range reported for the hybrid additive–deformation aluminum systems [48,52,63]. The intense plastic deformation and thermal–mechanical coupling during FSP produced a more refined equiaxed grain structure with an increased boundary density and uniform crystallographic orientation. Moreover, the equiaxed and more homogeneous microstructures produced by the FSP minimize the anisotropy, enhance the uniform mechanical response, and reduce the likelihood of microcrack initiation.

**Figure 11:**
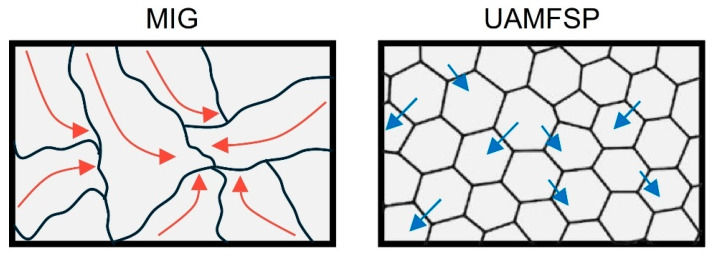
The schematic illustration of the dislocation-mediated strengthening in the MIG and the UAMFSP walls. In the MIG condition, coarse, irregular grains permit long dislocation mean free paths (red arrows), enabling easy glide and a low hardness. In the UAMFSP condition, the SPD during FSP produced fine equiaxed grains, with a high boundary density, inducing dislocation pile-ups (blue arrows) at the grain boundaries and therefore significantly increasing the strength via Hall–Petch strengthening.

Figure 11 shows the dominant strengthening mechanism. As illustrated, in the MIG wall, coarse dendritic grains allowed long dislocation glide paths (red arrows), resulting in a low hardness of about 52 HV. On the other hand, in the UAMFSP wall, with a hardness value of about 76 HV, the ultrafine equiaxed grains generate dense dislocation pile-ups (blue arrows) on high-angle boundaries, which can increase the resistance to slip, a direct manifestation of Hall–Petch strengthening.

Comparable hardness and strength have been reported for hybrid FSP-assisted WAAM fabricated aluminum alloys, where the interlayer deformation transformed columnar grains into fine equiaxed morphologies and enhanced both the yield strength and fatigue endurance [53,63]. For example, Zhou et al. [48] reported that Al–Cu–Sc alloys processed by the FSP-assisted WAAM process exhibited substantial yield and ultimate strength gains of about 30% compared to as-deposited walls. Also, Sun et al. [52] similarly demonstrated that an interlayer FSP can eliminate the metallurgical defects, such as porosity, refine grains, and redistribute Al_2_Cu precipitates, collectively improving the tensile strength and ductility while also promoting a more homogeneous behavior.

Collectively, the results of the mechanical performance evaluation using microhardness measurement confirm that the UAMFSP process can successfully overcome the characteristic limitations of the conventional WAAM process by producing fine-grained, more homogeneous walls with enhanced strength and improved reliability.

It should be noted that the mechanical assessment in this work is limited to microhardness measurements, which reflect local resistance to plastic deformation and support relative strengthening trends; comprehensive validation of bulk strength, ductility, fatigue performance, and anisotropy requires additional macroscale testing (e.g., tensile, fatigue, and compression) to fully validate bulk behavior and anisotropy.

While interlayer FSP can improve microstructural uniformity, it may also redistribute residual stresses due to severe plastic deformation and local thermal input, and the net stress state should be verified using dedicated residual-stress measurements in future work. In addition, the UAMFSP route introduces an extra processing step that can increase manufacturing time and may present scalability challenges for thicker multilayer builds; therefore, throughput and process planning (e.g., layer-to-layer scheduling and toolpath strategy) should be assessed alongside mechanical testing in follow-on studies.

Future work will include cross-sectional microhardness line scans and/or 2D hardness mapping to quantify local gradients between the deposited region, the stirred zone, and adjacent material.

### 4.4. Implications and Final Remarks

The earlier presented comparative findings highlight that the processing strategy plays a vital role in governing the process–structure–property relationships of aluminum alloy walls fabricated using MIG and the hybrid UAMFSP routes. In the MIG wall, the dominance of course, irregular, and partially dendritic grains reflected the cumulative effects of interpass heat accumulation and slow solidification. These structural characteristics can foster the anisotropy [6,7], localized stress concentrations and the nonuniform mechanical behavior, which collectively can degrade the toughness, fatigue life, and strength. These limitations are consistent with those of prior studies on the WAAM process, which reported that uncontrolled thermal cycling and remelting can lead to uniform and heterogeneous microstructures, high residual stresses, and premature fatigue cracking [19,59]. In contrast, the UAMFSP-fabricated wall exhibited refined, equiaxed, and more homogeneous microstructure accompanied by significantly lower and more uniform peak temperatures.

These thermal and structural refinements are reflected in higher and more uniform microhardness (45.8% increase), indicating improved local resistance to plastic deformation; however, bulk mechanical validation (e.g., tensile and fatigue) remains outside the scope of the present study. The hybrid WAAM construction exploits the geometric freedom of additive processes while retaining the mechanical robustness of conventional manufacturing [60]. This principle underpins the UAMFSP strategy, which couples layer-wise deposition with solid-state deformation to achieve the design flexibility and structural reliability of the fabricated parts. This enhancement can be ascribed to Hall–Petch strengthening [54,64] arising from the reduced grain size in combination with the more homogeneous and defect-minimized microstructure achieved by the FSP. Such simultaneous refinement and homogenization are consistent with earlier hybrid FSP-assisted WAAM investigations on different aluminum alloys, which reported parallel improvements in hardness, yield strength, and fatigue endurance [19,52,63].

Overall, the results confirm that coupling MIG deposition with FSP overcomes the limitations of conventional WAAM and establishes a robust process–structure–property relationship. These outcomes are aligned with our group’s patented vision for hybrid manufacturing [20], which integrates deposition, deformation, and machining into a single thermomechanically balanced workflow to achieve reproducible, more homogeneous, and high-performance components.

Collectively, these findings establish the UAMFSP approach as a robust, scalable, and thermomechanically balanced pathway to fabricate aluminum alloy components that need high structural integrity, more homogeneous microstructure, and reliability, which are key prerequisites for aerospace, marine, and automotive applications, where the conventional WAAM is constrained by anisotropy and heat-accumulation effects [60].

## 5. Conclusions

This study demonstrates that the processing strategy strongly influences the thermal response, microstructure, and local hardness trends of Al 4043 walls produced by MIG-based WAAM and the hybrid UAMFSP process. However, any inferred improvements in bulk mechanical behavior or anisotropy-related performance based on microhardness and microstructural indicators should be considered preliminary within the scope of this process study. For a broader industrial adoption, future research should extend this approach into thicker multilayer builds, explore additional aluminum alloys and hybrid material systems, and investigate their performance under the service-relevant loading conditions, such as tensile, fatigue, and thermal cycling. Such a research study is essential for establishing the scalability, durability, and qualification potential of the UAMFSP as a reliable manufacturing route for the high-performance structural components used in aerospace, marine, and automotive applications.

Scientific conclusions of this study are: (1) UAMFSP reduces thermal accumulation relative to MIG-based WAAM, as evidenced by IR thermography trends across layers; (2) interlayer FSP transforms the as-deposited dendritic/columnar morphology into a refined, more homogeneous equiaxed microstructure, supported by quantitative grain-shape statistics; and (3) microhardness measurements indicate a significant increase and improved spatial uniformity after UAMFSP; however, these results reflect local property trends and do not constitute full mechanical validation.

The technological implications (process-focused) of the study are that the integrated UAMFSP workflow on an automated CNC platform demonstrates a practical route for coupling deposition and solid-state deformation to improve microstructural control during WAAM of Al 4043.

Future studies will include complete mechanical validation (tensile, fatigue, and thermal cycling), residual-stress measurements, and EBSD-based texture characterization. Additionally, scalability will be assessed through taller/thicker multilayer builds and additional aluminum alloys/hybrid material systems, alongside evaluation of manufacturing time, throughput, and process robustness to support industrial viability.

## Figures and Tables

**Table 1:** Nominal chemical compositions of the substrate (AA6061 aluminum alloy) and the filler (ER4043 aluminum wire) in wt.%.

Element	Mg	Fe	Mn	Cr	Si	Cu	Zn	Ti	Al
Substrate	0.8–1.2	≤0.70	≤0.15	0.04–0.35	0.40–0.80	0.1–0.4	≤0.25	≤0.15	Bal.
Filler (ER4043)	≤0.05	≤0.8	≤0.05	-	4.5–6.0	≤0.3	≤0.1	≤0.2	Bal.

**Table 2:** MIG parameters used for the fabrication of the two walls.

Voltage (U)	Current (I)	Welding Speed (*v*) (mm/min)	Wire Stick-Out (mm)	Argon Flow Rate (CHF)
18	120	330	9	27

**Table 3:** FSP parameters used in the fabrication of the UAMFSP wall.

Layer	Tool Rotational Speed (*vs*) (RPM)	Tool Travel Speed (*v*) (mm/min)	Plunge Depth (mm)
1	600	50	0.2
2 & 3	1200	50	0.2

**Table 4:** Grain size and morphology statistics of MIG and UAMFSP walls.

Wall	Ā (µm^2^)	Ã (µm^2^)	σA	$\bar{P}$(µm)	$\tilde{P}$	σP	$\bar{D}$	$\tilde{D}$	σD	$\bar{R}$	$\tilde{R}$	σR
MIG	313.6	219.8	335.2	73.4	68	49.6	17.2	16.7	10.2	0.71	0.61	0.38
UAMFSP	10.9	6.4	13.1	14	12.1	9.5	3.2	2.9	1.9	0.62	0.57	0.29

Note: A = Grain Area; P = Grain Perimeter; Deq = Grain Equivalent Diameter; R = Grain Roundness. Overbar = mean; tilde = median; σ = standard deviation.

**Table 5:** The average of maximum temperatures (°C) at Layers 1–3 for the MIG and UAMFSP walls.

Process	L1	L2	L3
MIG	897.63	869.42	998.15
UAMFSP	324.45	386.21	277.23

## Data Availability

The raw data supporting the conclusions of this article will be made available by the authors on request.

## Abbreviations

The following abbreviations are used in this manuscript:

AA	Aluminum Alloy
AM	Additive Manufacturing
BGS	Bimodal Grain Structure
CDRX	Continuous Dynamic Recrystallization
DED	Directed Energy Deposition
DEDAM	Direct Energy Deposition Additive Manufacturing
DOE	Design of Experiments
DRX	Dynamic Recrystallization
EBSD	Electron backscatter diffraction
EDS	Energy-dispersive X-ray spectroscopy
ER4043	Al–Si welding wire/filler alloy (AWS designation)
FSP	Friction Stir Processing
FSW	Friction Stir Welding
HV	Vickers Hardness
IR	Infrared
MIG	Metal Inert Gas
PAW	Plasma Arc Welding
SPD	Severe Plastic Deformation
TIG	Tungsten Inert Gas
UAMFSP	Unified Additive–Deformation Manufacturing Process
WAAM	Wire Arc Additive Manufacturing
Symbols	
σA	Standard deviation of grain area (µm^2^)
σDeq	Standard deviation of equivalent diameter (µm)
σP	Standard deviation of grain perimeter (µm)
σR	Standard deviation of grain roundness
A	Grain area (µm^2^)
Ā	Mean grain area (µm^2^)
Ã	Median grain area (µm^2^)
Deq	Grain equivalent diameter (µm)
$\bar{D}$	Mean equivalent diameter (µm)
$\tilde{D}$	Median equivalent diameter (µm)
ε	Emissivity
HV	Vickers microhardness
I	Welding current (A)
L1, L2, L3	Layer 1, Layer 2, Layer 3 (build sequence)
P	Grain perimeter (µm)
$\bar{P}$	Mean grain perimeter (µm)
$\tilde{P}$	Median grain perimeter (µm)
R	Grain roundness
$\bar{R}$	Mean grain roundness
$\tilde{R}$	Median grain roundness
T	Temperature field at position (°C)
U	Welding voltage (V)
*v*	Tool/substrate travel speed (mm·min^−1^)
